# Cerebrospinal fluid findings and hypernatremia in COVID-19 patients with altered mental status

**DOI:** 10.1186/s12245-020-00327-4

**Published:** 2020-12-09

**Authors:** Hale Toklu, Latha Ganti, Ettore Crimi, Cristobal Cintron, Joshua Hagan, Enrique Serrano

**Affiliations:** University of Central Florida College of Medicine Department of Clinical Sciences, North Florida Regional Medical Center, GME Bldg., Suite 122E, 1147 NW 64th Terrace, Gainesville, FL 32605 USA

**Keywords:** CSF, Cerebrospinal fluid, Hypernatremia, Sodium, COVID-19, SARS-CoV2

## Abstract

**Background:**

The objective of the study was to assess the cerebrospinal fluid (CSF) findings in COVID-19 patients.

**Aims:**

This was an observational retrospective cohort from electronic medical records of hospitalized patients (*n* = 2655) with confirmed COVID-19 between February 15, 2020, and April 15, 2020, in 182 hospitals from a large health system in the USA. The review of data yielded to a total of 79 patients in 20 hospitals who had CSF analysis.

**Methods:**

Outcomes during hospitalization, including hospital length of stay, disease severity, ventilator time, and in-hospital death were recorded. Independent variables collected included patient demographics, diagnoses, laboratory values, and procedures.

**Results:**

A total of 79 patients underwent CSF analysis. Of these, antigen testing was performed in 73 patients. Ten patients had CSF analysis for general markers such as total protein, cell count, glucose, clarity, and color. Seven of the 10 cases (70%) had normal total cell count and normal white blood cell count in CSF. Sixty-three percent (5/8) had elevated total protein. Two patients had normal levels of lactate dehydrogenase (LDH) and 1 patient had significantly elevated (fourfold) neuron-specific enolase (NSE) level in CSF.

**Conclusion:**

Unlike bacterial infections, viral infections are less likely to cause remarkable changes in CSF glucose, cell count, or protein. Our observations showed no pleocytosis, but mild increase in protein in the CSF of the COVID-19 patients. The fourfold elevation of NSE may have diagnostic/prognostic value as a biomarker in CSF for COVID-19 patients who have altered mental status.

**Graphical abstract:**

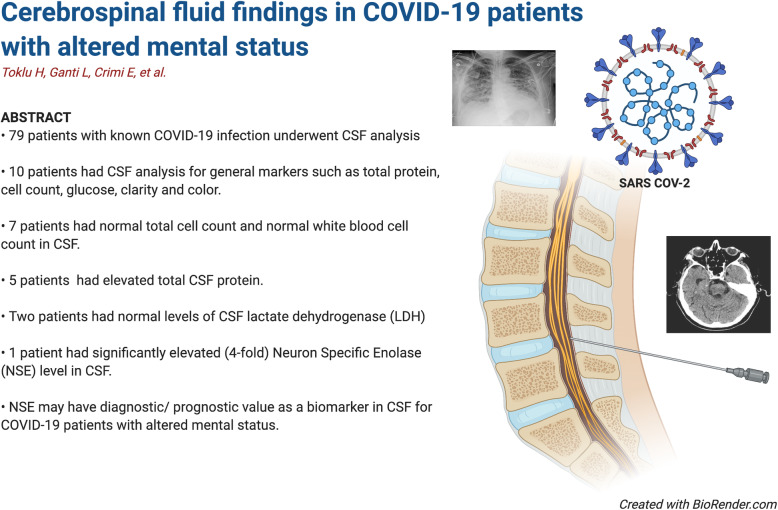

## Introduction

The coronaviruses are RNA viruses that are responsible for zoonotic infections. The strains of coronaviruses that have caused outbreaks in recent history include the systemic acute respiratory syndrome by SARS-CoV and the Middle East respiratory syndrome by MERS-CoV. SARS-CoV was the causal agent of the severe acute respiratory syndrome outbreaks in 2002 and 2003 in China, while MERS-CoV was the responsible agent for the outbreak in 2012 in Saudi Arabia [1]. Coronavirus disease 2019 (COVID-19) is an infectious disease caused by a new strain of coronavirus type 2, SARS-COV-2 [2].

COVID-19 presents with various symptoms ranging from mild to acute respiratory distress syndrome (ARDS), as well as gastrointestinal symptoms, cardiovascular, and neurologic symptoms. There are worldwide reports of neurological symptoms associated with COVID-19 [3–14]. Central nervous system manifestations were reported between 25 and 57% with less frequent symptoms of neuropathy and musculoskeletal signs. These symptoms vary from encephalitis, encephalopathy, acute demyelination, acute cerebrovascular events, altered mental status, impaired consciousness, dizziness, ataxia, seizures, hypogeusia, hyposmia, neuropathic pain, and headache.

In critically ill patients with neurological symptoms, the CSF analysis helps differential diagnosis and can serve as a marker of severity and prognosis, especially in bacterial infections. The CSF findings are also valuable in acute inflammation and demyelination [15]. In addition to the analysis of general components of CSF, antigen, antibody, and other biomarker tests can be valuable for prognosis. Several biomarkers such as S100B, neuron-specific enolase (NSE), and glial fibrillary acid protein (GFAP) were shown to be associated with the clinical outcome in patients with head concussion trauma, ischemic stroke, intracerebral hemorrhage, cardiac arrest, anoxic encephalopathy, encephalitis, brain metastasis, and status epilepticus [16–20].

In this observational cohort study, we evaluated the cerebrospinal fluid findings in hospitalized patients with confirmed COVID-19.

## Methods

### Study design

This observational retrospective cohort study was conducted through the electronic medical records (EMR) of 182 hospitals of large health system across the USA. The EMR of hospitalized patients (*n* = 2655) with confirmed novel coronavirus disease 2019 (COVID-19) between February 15, 2020, and April 15, 2020, were reviewed. The data extraction yielded to a total of 79 patients in 20 hospitals who had encephalopathy and underwent CSF analysis.

### Inclusion criteria

Patients who were hospitalized and had confirmed positive COVID-19 (ICD10 U07.1) were included in this study. Patients were considered to have confirmed COVID-19 infection if the initial nasopharyngeal swab result was positive for SARS-CoV-2 by the polymerase chain reaction (PCR) testing.

### Main outcomes and measures

Outcomes included hospital length of stay, disease severity, ventilator time, and in-hospital death. Patient demographics, diagnosis labs, and procedures were also reviewed.

### Ethics

This study was conducted in accordance with the Declaration of Helsinki and approved by the HCA Institutional Review Board (IRB) Manager system (Protocol no: 2020-551). The requirement for written informed consent was waived as the obtained data was de-identified.

The data that support the findings of this study are available from HCA but restrictions apply to the availability of these data, which were used under license for the current study, and so are not publicly available. Data are however available from the authors upon reasonable request and with permission of HCA.

### Statistical analysis

The frequency analysis was performed using the IBM SPSS Statistics software for Windows version 24 (IBM Corp, Armonk, NY, USA).

## Results

**Fig. 1:**
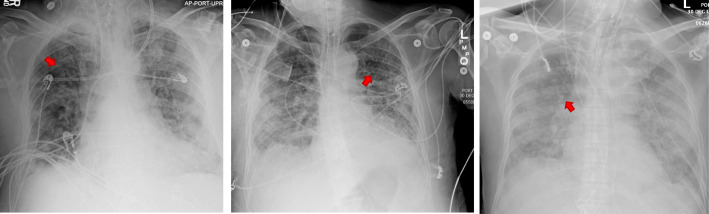
Chest X-rays of the three patients with COVID-19. Red arrows indicate the ground-glass opacities

**Fig. 2:**
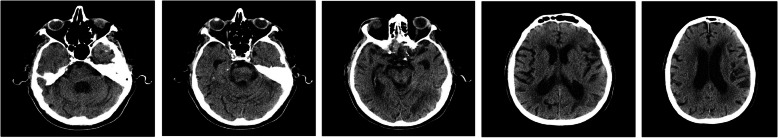
Magnetic resonance imaging (MRI) of the brain

A total of 79 patients in 20 hospitals had CSF analysis. The mean age was 65 ± 15 years [range 25-90; median 68 years]. Seventy-five percent of the patients were 55 years old and above. Sixty-three percent were male. Twenty-four percent of the patients presented neurological symptoms such as encephalopathy, altered mental status, impaired consciousness, dizziness, ataxia, myoclonus, unspecified convulsions, loss of taste or smell, facial weakness nausea, vomiting, aphasia, dysphasia, and lack of coordination. Sixty-six percent stayed in the hospital for more than 3 days and 35% required ventilator support for more than 72 h (Table 1). The chest x-ray and brain MRI are shown in Figs. 1 and 2.

**Table 1:** Demographics of the patients who had a cerebrospinal fluid (CSF) analysis. *N* = 79

		*N*	(%)
Age (years)	< 55	20	25.3
	≥ 55	59	74.7
Sex	Male	50	63.3
	Female	29	36.7
Race	White	46	58.2
	Black or African American	22	27.8
	Asian	2	2.5
	Other	9	11.4
Hypernatremia	Yes	20 (4 of them persistent)	25.3 (20% of hypernatremia cases were ≥ 48 h)
	No	59	74.7
Disease severity	Mild-moderate	37	46.8
	Severe-critical	42	53.2
In-hospital mortality	Ex	19	24.1
	Survivor	60	75.9
Hospital length of stay	≥ 1 week	27	34.2
	< 1 week	52	65.8
Ventilator time	≥ 72 h	28	35.4
	< 72 h	3	3.8
	None	48	60.8

Twenty of these patients had hypernatremia and 4 (20%) of these had persistent hypernatremia for ≥ 48 h.

The CSF was used for testing the presence of bacterial or viral antigens in 73 patients. The primary antigen testing was for Streptococcus pneumonia (74%) followed by adenovirus (17.8%). Ten patients had CSF analysis for proteins, cell count, glucose, and general appearance (Table 2).

**Table 2:** Cerebrospinal fluid (CSF) analysis. *N* = 79 patients

	Type/detail	***N*** (%)	Result
***Pathogen antigen/antibody***	Streptococcus pneumonia	54 (74.0)	99% negative 1 positive for Streptococcus pneumonia 1 false positive for West Nile virus
	Adenovirus ADV	13 (17.8)	
	*Cryptococcus* sp.	3 (4.1)	
	Herpes simplex virus HSV	3 (4.1)	
	VDRL	2 (2.7)	
	Cytomegalovirus	2 (2.7)	
	Enterovirus	2 (2.7)	
	Escherichia col	2 (2.7)	
	Varicella zoster	2 (2.7)	
	Influenza	2 (2.7)	
	West Nile	2 (2.7)	
	Total	73 patients (97 results)	
***Antibody***	Immunoglobulin G	2	1 normal +1 high
***LDH***	Lactate dehydrogenase	2	normal
***NSE***	Neuron specific enolase	1	Very high (fourfold)
***CSF analysis***	Clarity	8	7 clear +1 cloudy
	Color	8	7 colorless +1 red
	Glucose	8	7 high
			1 normal
	Total protein	8	5 high
			3 normal
	Total cell count	10	3 high
			7 normal
	WBC	10	4 high
			6 normal
***Total***		79 patients (130 results)	

*ADV* adenovirus; *HSV* herpes simplex virus; *LDH* lactate dehydrogenase; *NSE* neuron-specific enolase; *VDRL Venereal Disease Research Laboratory*; *WBC* white blood cells; *IRB* Institutional Review Board

## Discussion

Unlike bacterial infections, viral infections are less likely to cause remarkable changes in CSF glucose, cell count, or protein [15, 21]. However, there is increasing evidence about the neuroinvasive potential of SARS-CoV2 in COVID-19 resulting in disseminated encephalomyelitis and encephalopathies [22–27]. One hypothesis is that the respiratory failure could be partially due to the neuroinvasion of the brain issue—particularly brain stem and medulla oblongata [28]. The virus uses ACE2 to enter the cell, which is abundantly present in the capillary endothelium of the cerebral tissue [29]. The infection of SARS-CoV has been reported in the brains from both patients and experimental animals, where the brainstem was heavily infected [28]. However, the researchers from the USA could not detect SARS-CoV2 antigen in the CSF of two-stroke cases, which raises the doubt about blood-brain barrier disruption theory in COVID-19 infections [30]. Another paper also failed to detect the virus antigen in a patient with encephalopathy despite the marked increase in the interleukins predominantly IL-10 [31]. Hence, a study from Brazil reported a demyelinating disease in which the viral genome of SARS-CoV was detected in CSF [32]. Another surprising finding was that the virus RNA was detected in CSF but not in the nasopharyngeal swab taken from a patient with meningitis/encephalitis in Japan [27]. The same phenomenon was seen in a 20-year-old patient in Ireland [33].

A recent paper from the USA reported increased IgM and cytokine levels in the CSF of three patients with encephalopathy and encephalitis [23]. Increased protein and IgG but normal cell count was observed in a patient from Switzerland who had cerebral microbleeds, and focal EEG changes associated with COVID-19 [34] (Fig. 1). On the other hand, there was no significant change in the CSF of a COVID-19 patient with encephalitis in Wuhan. The total protein, WBC count, and glucose were within the normal range and they were not able to detect anti-SARS-CoV2 IgM and IgG in CSF [25]. Normal CSF findings were also reported for other two cases from Iran [22, 35]. Another 2 cases from France and 2 from the USA also revealed normal cytology and elevated protein and glucose [36, 37]. Consistent with these reports, our small cohort showed that 7 of the 10 cases (70%) had normal total cell count and normal WBC count in CSF. Sixty-three percent (5/8) had mildly elevated total protein. Additionally, two patients had normal levels of LDH and 1 patient had significantly elevated neuron-specific enolase in CSF, which is an indicator of neuronal damage. Supporting our findings, the CSF analysis of a patient from Sweden with COVID-19-related acute necrotizing encephalopathy, showed only a slight increase in protein and monocytes, while the levels of neuronal injury markers such as tau, NfL (neurofilament light), and GFAP were extremely high [24]. Based on the observation from our small cohort, we found that COVID-19 encephalopathy does not cause remarkable changes in CSF in terms of cell count and protein amount, but an increase in neuronal injury biomarker NSE (Fig. 2).

Another interesting observation was that our 6 of the 8 cases had hypernatremia, which was persistent in 4 cases lasting for more than at least 48 h. Our observation with hypernatremia has also been reported recently by other researchers [38]. They observed treatment-resistant hypernatremia in 6 of their 12 critically ill patients who required mechanical ventilation. There did not find any correlation between plasma sodium concentrations and sodium input. However, plasma chloride was elevated. This elevation was accompanied by a decrease in potassium, which was consistent with abnormally increased renal sodium reabsorption [38]. It is known that prolonged hypo- and hypernatremia may contribute to encephalopathy and osmotic demyelination and is associated with increased mortality [39]. As an indicator of neuronal injury, NSE increase in CSF in Guillain-Barre syndrome was shown in earlier studies [40]. Seven of our 8 patients also had severe sepsis resulting in multi-organ failure. Six of our 8 cases expired and the average length of stay in hospital for these 8 was 30 days. Furthermore, all of them required ventilator support varying between 5-28 days. The laboratory work revealed sepsis and morbidities associated with sepsis (Table 3). It is well-known that the metabolic changes in sepsis, particularly sodium imbalance may result in demyelination and disruption of blood-brain barrier which enables the entrance of the pathogen to the central nervous system.

**Table 3:** The clinical profile of patients who had a CSF analysis. *N* = 8

*Hypernatremia*								*Normal sodium*	
		***Case 1***	***Case 2***	***Case 3***	***Case 4***	***Case 5***	***Case 6***	***Case 7***	***Case 8***
*Demographics*	Age (years)	79	66	76	77	58	82	64	64
	Sex	M	M	M	M	M	M	M	M
	Race	White	Asian	Asian	White	White	White	White	White
*Serum* (*highest*)	White blood cell count (#/ul)	10.0	23.1	31.4	13.63	19.8	10.3	45.9	18.2
	Hemoglobin (g/dl)	14.9	15.2	13.4	16.7	14	11.7	14.7	12.6
	D-dimer (mg/L FEU)	3.768	31.04		1.62		2.60	36.39	
	Alanine aminotransferase (IU/L)	34	54	157	95	164	75	93	36
	Aspartate aminotransferase (IU/L)	99	65	122	91	226	62	91	77
	Creatinine (mg/dL)	1.21	1.18	7.27	2.61	2.85	1.41	7.82	10.60
	Troponin I (ng/mL)	0.036	< 0.020	0.210	0.018	0.352	0.150	1.110	0.238
	Sodium (mEq/L)	163	152	146	156	150	163	142	145
	Hypernatremia severity • Mild (146-150 mEq/L • Moderate (151-154 mEq/L) • Severe (≥ 155 mEq/L)	Severe	Moderate	Mild	Severe	Mild	Severe	None	None
*Vitals* (*admission*)	SpO_2_ (%)	?	89	93	91	90	92	93	88
	Temperature (F)	?	98.1	100.4	97.5	98.3	97.9	102.7	101.4
	Pulse (beats/min)	?	105	107	71	97	97	146	74
	Blood pressure (mmHg)	?	142/69	132/66	139/68	113/55	128/76	133/95	183/69
*Arrival mode to Emergency Department*			Walk-in	Ambulance			Ambulance	Walk-in	Walk-in
*Cerebrospinal Fluid* (*CSF*) *analysis*	White blood cell count (#/ul)	Normal	Normal	Normal	High	Normal	Normal	Normal	High
	Total cell count (#/ul)	Normal	Normal	High	High	Normal	Normal	Normal	High
	Total protein (mg/dl)	High	High	Normal	High	Normal	Normal	High	High
	Glucose (mg/dl)	High	Normal	High	High	High	High	High	High
	IgG		Normal		High				
	Neuron specific enolase (NSE)				High				
	Lactate dehydrogenase (LDH)		Normal	Normal					
*Clinical outcome*	Neurological symptoms		Myoclonus, metabolic encephalopathy, unspecified convulsions, anorexia	Metabolic encephalopathy, encephalitis and encephalomyelitis, unspecified convulsions	Nausea, seizures, encephalopathy		Cerebral infarction, quadriplegia, unspecified, febrile convulsions, dysphagia		Metabolic encephalopathy, absence epileptic syndrome, intractable, with status epilepticus
	Other diagnosis		Sepsis, ARDS, hypertension, benign prostate hypertrophy	Sepsis, ARDS, cardiac arrest, acute kidney failure, type 2 DM	Sepsis, ARDS, acute kidney failure, acute liver failure		Sepsis, ARDS, type 2 DM, diabetic neuropathy, nephropathy, acute diastolic heart failure	ARDS, Morbid obese, type 2 DM, HT	ARDS, type 2 DM, HL, cardiac arrest
	Disease severity	Severe/critical	Severe/critical	Severe/critical	Severe/critical	Severe/critical	Severe/critical	Severe/critical	Severe/critical
	Ventilator need	Yes	Yes	Yes	Yes	Yes	Yes	Yes	Yes
	Ventilator time	?	28 days	25 days	17 days	27 days	16	11	5
	Length of stay in hospital	26	41	45	20	39	20	31	17
	In-hospital mortality	Ex	Ex	Ex	Ex	Discharged	Ex	Discharged	Ex

## Conclusion

To our knowledge, this is the largest cohort to report the CSF findings in COVID-19 patients. However, further studies are needed to have a significant analysis to evaluate the association between CSF findings and clinical outcome.

## Data Availability

The data stored electronically and the datasets used and/or analyzed are available at the HCA GME Research repository on request.

## Abbreviations

ADV: Adenovirus
ARDS: Acute respiratory distress syndrome
COVID-19: Coronavirus disease 2019
CSF: Cerebrospinal fluid
EMR: Electronic medical records
GFAP: Glial fibrillary acid protein
HSV: Herpes simplex virus
IRB: Institutional Review Board
LDH: Lactate dehydrogenase
NfL: Neurofilament light
NSE: Neuron-specific enolase
PCR: Polymerase chain reaction
SARS: Severe acute respiratory syndrome
VDRL: Venereal Disease Research Laboratory
WBC: White blood cells
